# Phytohustil^®^ and root extract of *Althaea officinalis* L. exert anti-inflammatory and anti-oxidative properties and improve the migratory capacity of endothelial cells *in vitro*

**DOI:** 10.3389/fphar.2022.948248

**Published:** 2022-12-08

**Authors:** Gabriel A. Bonaterra, Johanna Schmitt, Kim Schneider, Hans Schwarzbach, Heba Aziz-Kalbhenn, Olaf Kelber, Jürgen Müller, Ralf Kinscherf

**Affiliations:** ^1^ Department of Medical Cell Biology, Anatomy and Cell Biology, University of Marburg, Marburg, Germany; ^2^ Bayer Consumer Health Division, Phytomedicines Supply and Development Center, Steigerwald Arzneimittelwerk GmbH, Darmstadt, Germany

**Keywords:** *Althaea officinalis* L., anti-inflammatory, anti-oxidative, endothelial cells, marshmallow, migration, Phytohustil^®^, ROS

## Abstract

**Introduction:** *Althaea officinalis* L.'s root extract (REA) has been used as a medicinal plant since ancient times to treat a cough. Applying REA leads to a protective film that induces a faster regeneration of the lesioned laryngopharyngeal mucosa caused by dry coughs. The buccopharyngeal mucosa is a highly vascularized tissue. In this regard, anti-inflammatory/-oxidant phytochemicals that improve the repair of the lesion site, e.g., neovascularization in the wound, are critical for promoting healing. For this reason, it is essential to investigate the effects of Phytohustil^®^ and REA on different cellular components of the mucosa under conditions similar to those found in the injured mucosa. Thus, this *in vitro* study investigated the anti-inflammatory/oxidative and pro-migratory properties of Phytohustil^®^ cough syrup on vascular endothelial cells.

**Methods:** Human umbilical vein endothelial cells (HUVEC) were pretreated (24 h) with Phytohustil^®^, its excipients, or REA, followed by incubation with hydrogen peroxide (H_2_O_2_; 1 h; pro-oxidative) or with lipopolysaccharides (LPS; 3 h; pro-inflammatory). Viability and cytotoxicity were measured by PrestoBlue^®^ assay. Intracellular reactive oxygen species (ROS) were quantified with 20-70-dichlorofluorescein diacetate (DCFDA). The release of interleukin 6 (IL6) was determined by enzyme-linked immunosorbent assay (ELISA). The migratory capacity of HUVEC was measured using a scratch assay.

**Results:** Our results show that Phytohustil^®^, its excipients and REA were not cytotoxic. Pretreatment of HUVEC (24 h) with Phytohustil^®^ or REA inhibited the LPS-activated IL6 release. Phytohustil^®^ or REA inhibited the H_2_O_2_-induced cytotoxicity and intracellular ROS production. Phytohustil^®^ and REA significantly stimulated wound closure compared to the control.

**Conclusion:** Our data show that Phytohustil^®^ and REA have anti-inflammatory/-oxidant properties and improve the migratory capacity of vascular endothelial cells. These properties may contribute to the healing characteristics of Phytohustil^®^ and support the benefit of Phytohustil^®^ in patient’s treatment of irritated oral mucosa.

## Introduction

Lesions of the oral and laryngopharyngeal mucosa have many etiologies, including viral or bacterial infections and local irritation due, e.g., to a dry cough. The use of phytochemicals in treating oral mucosa lesions has gained more attention in recent years in the international scientific community and has been reviewed recently (Salehi et al., 2019). Such a phytopharmaceutical is Phytohustil^®^ cough syrup which is used for the symptomatic treatment of irritations of mucous membranes caused by dry and irritable coughs. Phytohustil^®^ contains the dried root extract of *Althaea (A.) officinalis* L. (REA). *A. officinalis* L., has been used since ancient times to treat the irritation of oral, pharyngeal, or gastric mucosa (Schmidgall et al., 2000; Sendker et al., 2017). Their pharmaceutical use involves forming a layer that protects the epithelial membranes. The standard oral use of REA is associated with the adhesive properties of the polysaccharides to the epithelial mucosa, which protects from mechanical injuries and microbial invasion (Sendker et al., 2017).

Skin and oral mucosa are similar in morphology and functions, but there are differences related to homeostatic conditions (Waasdorp et al., 2021). Wound healing is a close-fitting regulated process to recover tissues after damage. Compared to the skin, the oral mucosa is lined by a nonkeratinized stratified squamous epithelium. It contains the same cell types, e.g., endothelial cells, fibroblasts, keratinocytes, macrophages, etc., as the skin (Turabelidze et al., 2014). The lamina propria includes, e.g., connective tissue, blood vessels, neural elements (Squier and Kremer, 2001), and fibroblasts. Characteristic of the oral mucosa is also a high vascularization and permeability (Novak et al., 2008) to supply the region with nutrients, immune cells, and oxygen. Angiogenesis, i.e., the formation of new vessels, is central to increased numbers of blood vessels at later stages of wound healing (Van der Veer et al., 2011). In this regard, phytochemicals that improve neovascularization and vascular maturation in the wound are essential to promote healing. Applying an anti-inflammatory and antioxidant drug that promotes cell migration, angiogenesis, etc., in the location of the damage may support wound healing. We have recently reported the effects of the anti-inflammatory, antioxidant, and pro-migratory properties of Phytohustil^®^ on human macrophages (Bonaterra et al., 2020). Many studies confirm that inflammation and oxidative stress occur after injury and are interdependent processes. In this context, REA exhibited strong total antioxidant activity, reducing power and free radical/superoxide anion radical scavenging (Elmastas et al., 2004). An antitussive effect of marshmallow root extract was found in an animal study using cats (Nosalova et al., 1993). In this regard, Phytohustil^®^ cough syrup an herbal medicinal product containing REA is widely used to treat irritations in the oral mucosa and throat.

Nevertheless, the effectiveness of REA is not only due to the mechanical protective effects of high molecular weight polysaccharides (Sendker et al., 2017) but also other components with healing properties. According to the experiments carried out on macrophages, as we have most recently published (Bonaterra et al., 2020), the objective of this study was to continue our previous research about the effects of Phytohustil^®^ on the different cellular components that make up the laryngopharyngeal mucosa. Therefore, this study has been aimed to investigate the anti-inflammatory, anti-oxidative, and pro-migratory properties of the phytopharmaceutical product Phytohustil^®^ cough syrup on endothelial cells. For this purpose, we have used primary human endothelial cells as an *in vitro* model of one of the vascular components of the mucosa.

## Materials and methods

### Cell culture

*In vitro* experiments were performed using commercial human umbilical vein endothelial cells (HUVEC) Cat.-No.: 121 0113; Provitro AG, Berlin, Germany), cultured in endothelial cell proliferation medium, basal (ECPM) according to the manufacturer’s instructions (Cat.-Nr.: 201 0001; Provitro AG) and supplements [Supplement-Mix (Cat.-Nr.: 218 0001) with antibiotics (Cat.-Nr.: 236 0350), Provitro AG]. No special permission regarding ethics approval for this study is required to use cells of human origin.

### Substances under test

The active constituent of STW42-H (REA) was provided by Steigerwald Arzneimittelwerk GmbH (Darmstadt, Germany). The root extract of *A. officinalis* (according to Kew Medicinal plant names service, https://mpns.science.kew.org/) was prepared as described previously (Bonaterra et al., 2020). Briefly, roots of *A. officinalis* were macerated in purified water with a drug-extract-ratio (DER) of 3-9:1 after drying. Phytohustil^®^ (100 g) cough syrup (Bayer AG, Leverkusen, Germany; batch-No. 730041) contains 35.6 g of the active ingredient liquid REA at a DER of 19.5-23.5:1 according to DAC (German Drug Codex, 1999). The excipients, ethanol, propyl-4-hydroxybenzoate, methyl-4- hydroxybenzoate and sucrose were provided by Steigerwald Arzneimittelwerk GmbH. The concentration of Phytohustil^®^ was expressed in µg/ml dry extract. A representative chromatogram of the REA batch-No. 14-0450 has been recently published (Bonaterra et al., 2020). REA was characterized and published previously (Sendker et al., 2015; Sendker et al., 2017; Fink et al., 2018). Diclofenac sodium salt CAS, 15307-79-6 (Merck KGaA, Darmstadt, Germany) was used as an anti-inflammatory control substance. The extract used in the current study is the active component in a commercially available preparation, registered in several countries as a medicinal herbal product. Therefore, the wild collection of the monographed plant, including botanical verification of the plant material by specialists according to the requirements of the European Pharmacopeia was performed. For this reason, the deposition of a specimen of the monographed plant is not deemed necessary.

### Measurements of the viability and cytotoxicity of human umbilical vein endothelial cells

HUVEC (5 × 10^3^) seeded in 100 µl ECPM/well in 96-well plates (Falcon™, BD Bioscience, Heidelberg, Germany) were incubated overnight. Afterward, the medium was changed, different concentrations (100, 500, and 1,000 µg/ml) of the REA, Phytohustil^®^, or its excipients, diluted in ECPM, were added and then the cells were treated (24 h) with the substances. HUVEC cultured only in ECPM were used as untreated control. Cell viability was assessed by using PrestoBlue^®^ reagent (Fisher Scientific GmbH, Schwerte, Germany), directly added to the ECPM at a final concentration of 10% and afterward, the optical density (OD) was measured at 570 nm and reference 600 nm using a Sunrise microplate reader (Tecan, Salzburg, Austria) (Bonaterra et al., 2020). Thereafter, the cells were fixed with 4% PFA in PBS and stained with crystal violet (CV) solution (0.04% crystal violet in 4% ethanol [v/v]) (Merck KGaA) and washed; subsequently, the cells were lysed in a 1% sodium lauryl sulfate, (SDS, Merck KGaA) solution (Bonaterra et al., 2020). The OD was measured at 595 nm and reference 655 nm to determine the total cell number. Results were expressed in % of viability measured by PrestoBlue^®^ ({[OD_570 nm/600 nm_ of samples] × 100%]}/OD_570 nm/600 nm_ of untreated control) or cytotoxicity 100%-({OD_570 nm/600 nm_ of samples] × 100%]}/OD_570 nm/600 nm_ of untreated control) measured by CV.

### Determination of anti-inflammatory effects

The release of human interleukin-1β (IL-1β), interleukin-6 (IL6), and tumor necrosis factor-α (TNF- α) was measured using a sandwich enzyme-linked immunosorbent assay (ELISA) (Bonaterra et al., 2020). After pretreatment for 24 h with different concentrations of REA, Phytohustil^®,^ or its excipients, HUVEC were activated with LPS 0.05 µg/ml or 0.1 μg/ml (3 h). Thereafter, the medium was harvested and centrifuged at 500 × g (5 min). The supernatant 100 µl/well was applied to Maxi-Sorb™ 96-well plates, which had previously been coated with 100 μl capture antibody (mouse anti-human IL-1β, IL6 or TNF- α, 4 μg/ml 1% BSA/PBS) overnight at 4°C. Afterward, the wells were washed with a wash solution consisting of 0.05% Tween-20/PBS. Concentrations of IL-1β, IL6, and TNF-α were determined using the DuoSet^®^ ELISA development kit (R&D Systems Europe, Ltd., Abingdon, United Kingdom) following the manufacturer’s instructions. Afterward, the cells were fixed with 4% PFA/PBS and stained with CV and the OD was measured as described above. The OD was measured at 490 nm and reference at 655 nm using a Sunrise microplate reader (Tecan). As described above, the concentrations of IL-1β, IL6, and TNF-α were calculated from respective standard curves and normalized with the total cell number determined by CV staining. Diclofenac sodium salt (Merck KGaA) was used as an anti-inflammatory control substance.

### Determination of the protective effects against H_2_O_2_-induced cytotoxicity

The protective effects of the REA, Phytohustil^®^ or its excipients against cytotoxicity induced by treatment with the pro-oxidant H_2_O_2_ were determined using the PrestoBlue^®^ (Fisher Scientific GmbH) as described above. In detail, 7.5 × 10^3^ HUVEC in 100 µl ECPM/well were seeded in 96-well plates (Falcon™, BD Bioscience). After overnight incubation, the medium was changed and the HUVEC were pretreated (24 h) with 1,000 μg/ml of the REA, Phytohustil^®^ or its excipients, followed by treatment (1 h) with H_2_O_2_.

### Determination of intracellular reactive oxygen species

Intracellular ROS was measured by using the fluorescent oxidant-sensitive probe 20-70-dichlorofluorescein diacetate (DCFDA, Merck KGaA). 9 × 10^3^ HUVEC were seeded in 100 µl ECPM/well using 96-well plates (Falcon™, BD Bioscience) and incubated overnight (Bonaterra et al., 2020). Afterward, the medium was changed, different concentrations of the REA, Phytohustil^®^, or its excipients, diluted in ECPM, were added and the cells were pretreated for 24 h with 100, 500, or 1,000 μg/ml REA, Phytohustil^®^, or its excipients. The next day 100 μl DCFDA (25 μM) were added to the cells in ECPM phenol red-free (-PR) containing 100, 200 or 300 µM H_2_O_2_ were added. Untreated HUVEC were used as a negative control, whereas HUVEC treated with H_2_O_2_ were used as a positive control, which was considered 100% intracellular ROS production. After adding H_2_O_2,_ fluorescence intensity was measured in relative fluorescence units (RFU) at different time points (0 min, 15 min, 30 min, 45 min, and 60 min). Total intracellular ROS was quantified at 495 nm excitation/529 nm emission using the BioTek Cytation™ 3 microplate reader (Agilent Technologies Germany GmbH, Waldbronn, Germany). Values in % fluorescence units (RFU) = [(sample DCFDA _495 nm_) x 100%]/(untreated DCFDA _495 nm_).

### Determination of the migratory capacity of human umbilical vein endothelial cells

The migratory capacity of HUVEC was determined using a scratch assay. Cells were seeded in 24-well plates and cultured until confluence was reached. A straight-lined scratch was applied to the cell monolayer with a pipette tip. Then the medium was changed twice to remove the damaged cells and debris. Afterward, the HUVEC were treated with 500 or 1,000 µg/ml of Phytohustil^®^, REA, or its excipients. The scratch was photographed at different time points (0 h, 4 h, 6 h–10 h) according to previous publications (Cao et al., 2010; Jonkman et al., 2014). It used an inverted microscope Axiovert 135 (Carl Zeiss AG, Oberkochen, Germany), equipped with a motorized stage and an AxioCam MRc camera (Carl Zeiss AG, Jena, Germany). Effects on HUVEC migration were plotted as a percentage of scratch closure (% SC = {[Δt] x 100%}/At 0 h; Δt = At 0 h–At 6 h where “At 0 h” is the area of the scratch measured immediately after scratching and “At 4 h or 6 h” are the area estimated at time 4 h or 6 h (Yue et al., 2010). Vascular endothelial growth factor (VEGF) was used as a positive control for migration.

### Statistical analyses

The SigmaPlot 12 software (Systat Software GmbH, Erkrath, Germany) was used to carry out statistical analyses by the unpaired Student’s *t*-test. When data failed normality and/or equal variance test Mann–Whitney *U*-test was used. Thus, the normality test (Shapiro-Wilk) and equal variance test were applied. Data shown as mean + SEM. *p* ≤ 0.05 was considered statistically significant (Bonaterra et al., 2020).

## Results

### Effect of Phytohustil^®^, its excipients or REA on the viability of human umbilical vein endothelial cells

**FIGURE 1:**
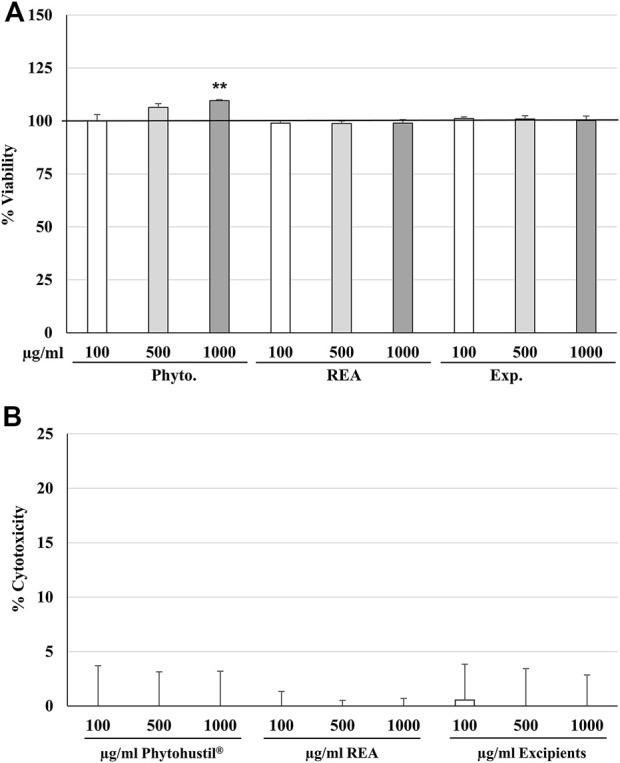
Effects on viability **(A)** or cytotoxicity **(B)** of HUVEC after 24 h treatment with Phytohustil^®^, its excipients, or root extract of *Althaea (A.) officinalis* L. (REA). Values in % viability of untreated control (100% viability) or in % cytotoxicity of control (0%) are given as mean + SEM; significance calculated by *t*-Test is indicated as **p ≤ 0.01 vs. untreated control (∼100% viability); *n* = 4–5 independent experiments.

We investigated the effects of treatment (24 h) of HUVEC with Phytohustil^®^, its excipients, or REA on the viability and cytotoxicity using the cell viability reagent Presto-Blue^®^ or CV cell cytotoxicity assay. Treatment (24 h) of HUVEC with different concentrations of Phytohustil^®^, its excipients or REA (100, 500, or 1,000 µg/ml), did neither affect their viability nor the cytotoxicity (Figures 1A,B), except 1,000 µg/ml Phytohustil^®^, which increased the viability by +9.6% significantly (*p* ≤ 0.01) (Figure 1A). Treatment of HUVEC with 0.05 or 0.1 µg/ml LPS did not affect their viability (not shown).

### Effects of Phytohustil^®^, its excipients or REA on IL-1β, IL6, and TNF-α release of human umbilical vein endothelial cells with or without lipopolysaccharides stimulation

**FIGURE 2:**
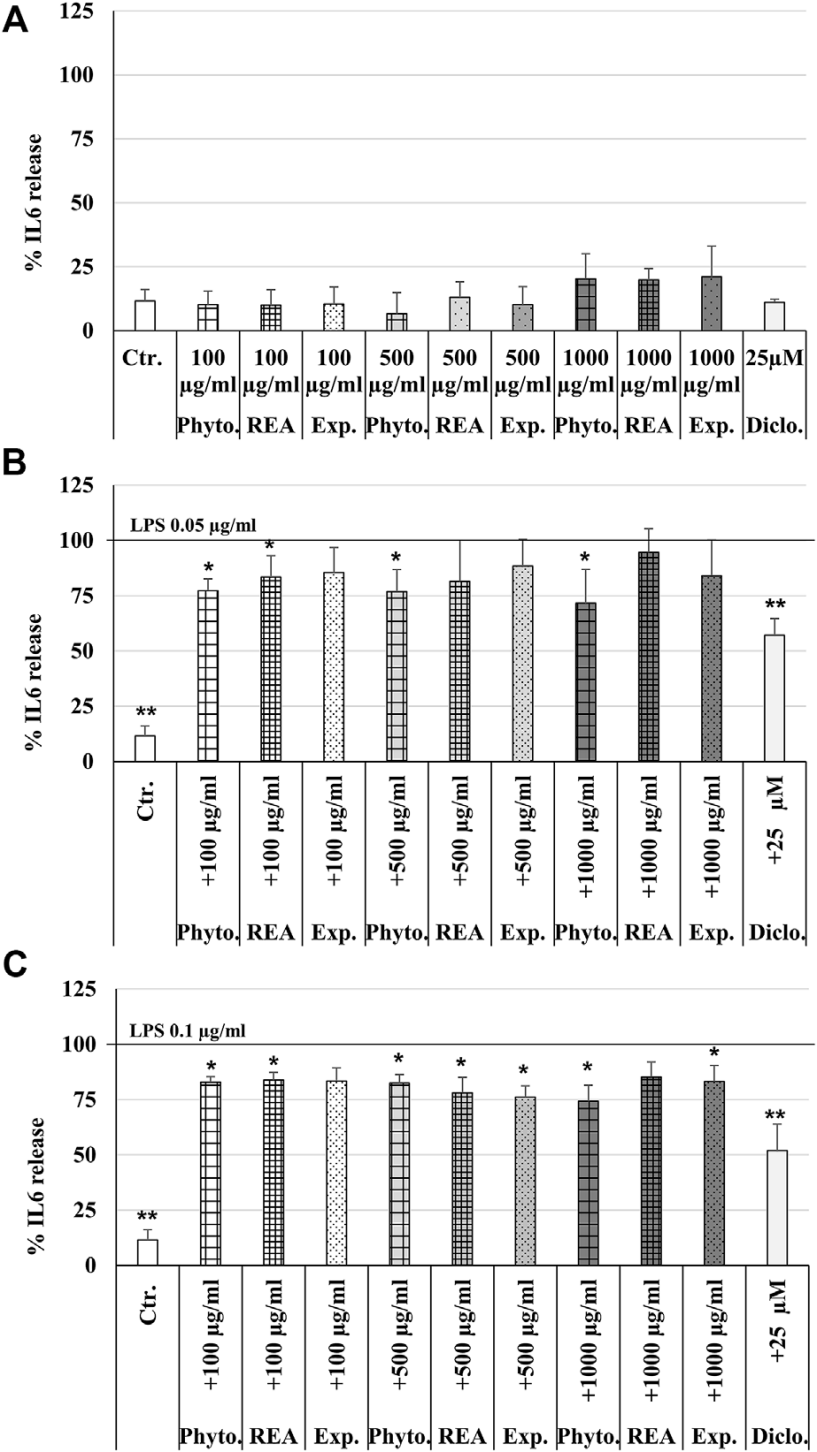
Effects of Phytohustil^®^ (Phyto.), its excipients (Exp.), or root extract of *A. officinalis* L (REA) alone on IL6 release **(A)**. Effects of pretreatment (24 h) of HUVEC with Phyto., REA or excipients and afterward 3 h LPS 0.05 µg/l **(B)** or 0.1 µg/ml **(C)** on IL6 release. Untreated control (Ctr. Med.). Data are given as mean + SEM; significance calculated by *t*-Test is indicated as *p ≤ 0.05 vs. LPS-treated HUVEC (100%); *n* = 4–8 independent experiments.

To investigate the anti-inflammatory properties, we determined the inhibition of the IL6 release of LPS-activated HUVEC. Treatment (24 h) of HUVEC with different concentrations (100 µg/ml, 500 µg/ml, 1,000 µg/ml) of Phytohustil^®^, its excipients, REA or diclofenac 25 µM alone did not affect the IL6 release compared to the untreated control (Figure 2A). Previous experiments were performed stimulating HUVEC with 0.1–10 µg/ml LPS to find concentrations that stimulate cytokine release. We found that IL-6 but neither IL-1β nor TNF-α release was sufficiently stimulated to detect a possible anti-inflammatory effect of the substances under test (data not shown). Incubation of HUVEC with LPS (0.05 or 0.1 µg/ml) alone induced a significantly (*p* ≤ 0.01) increased IL6 release of 88.4% compared to untreated control (Figures 2B,C). Furthermore, HUVEC were pretreated (24 h) with 100, 500, or 1,000 µg/ml Phytohustil^®^, its excipients, or REA and afterward activated for 3 h with LPS (0.05 or 0.1 µg/ml). Pretreatment with Phytohustil^®^ (100 µg/ml or 1,000 µg/ml) significantly (*p* ≤ 0.05) inhibited 0.05 µg/ml LPS-induced IL6 release by 22.7% or 28.4%. Diclofenac (25 µM) used as a positive anti-inflammatory control significantly inhibited the LPS effect by 42.8% (Figure 2B). Whereas pretreatment with 100 µg/ml Phytohustil^®^ or REA significantly (*p* ≤ 0.05) inhibited the IL6 release by 17.1% and 16.2% compared to treatment with 0.1 µg/ml LPS (Figure 2C). Pretreatment with 500 µg/ml of Phytohustil^®^, REA, or excipients significantly (*p* ≤ 0.05) inhibited the IL6 release by 17.5%, 22.0%, and 23.8% compared to HUVEC activated with 0.1 µg/ml LPS (Figure 2C). However, only the incubation with 1,000 µg/ml Phytohustil^®^ was able to significantly (*p* ≤ 0.05) inhibit the IL6 release by 25.8% compared to 0.1 µg/ml LPS alone (Figure 2C). Diclofenac (25 µM) inhibited the IL6 release by 48% (*p* ≤ 0.05) compared to 0.1 µg/ml LPS (Figure 2C).

### Effects of Phytohustil^®^ its excipients or REA against H_2_O_2_-induced cytotoxicity in human umbilical vein endothelial cells

**FIGURE 3:**
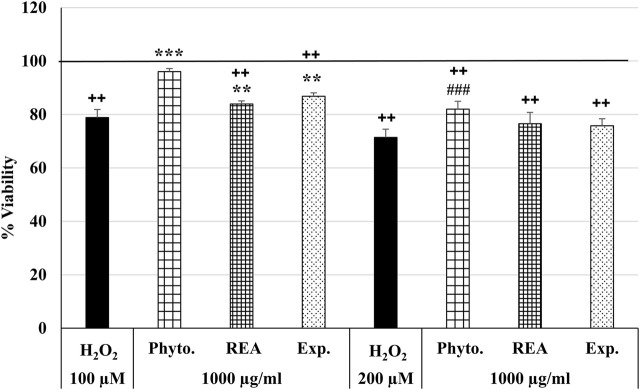
Effects of Phytohustil^®^ (Phyto.), its excipients (Exp.), or root extract of *A. officinalis* L. (REA) against H_2_O_2_–induced cytotoxicity. After pretreatment (24 h) of HUVEC with Phytohustil^®^, its excipients or REA and afterward 1 h with 100 µM or 200 µM H_2_O_2,_ the viability was measured by PrestoBlue^®^ assay. Values [in % viability of untreated control (100% viability)] are given as mean + SEM; significance calculated by *t*-Test is indicated as ***p ≤ 0.001 vs. 100 µM H_2_O_2_ or ^###^ *p* ≤ 0.001 vs. 200 µM H_2_O_2_ treatment; ^++^ *p* ≤ 0.01 vs. untreated control (100% viability); *n* = 6 independent experiments.

Furthermore, we were interested in investigating the anti-oxidative effects of Phytohustil^®^, REA, or excipients after H_2_O_2_ treatment. Thus, we first measured the cytotoxic effects of H_2_O_2_ on HUVEC and found that 100 µM or 200 µM H_2_O_2_ significantly (*p* ≤ 0.01) inhibited the viability by 21.1% and 28.5% compared to untreated control (∼100% viability) (Figure 3). Only pretreatment (24 h) of HUVEC with 1,000 µg/ml Phytohustil^®^ significantly (*p* ≤ 0.001) inhibited 100 µM H_2_O_2_ -induced cytotoxicity by 17.2% compared to 100 µM H_2_O_2_ and decreased the viability by 10.6% (*p* ≤ 0.001) compared to 200 µM H_2_O_2_ (Figure 3). Moreover, after pretreatment of HUVEC with 1,000 µg/ml Phytohustil^®^ the viability was similar to untreated control (Figure 3).

### Effects of Phytohustil^®^, its excipients or REA against H_2_O_2_-induced reactive oxygen species production

**FIGURE 4:**
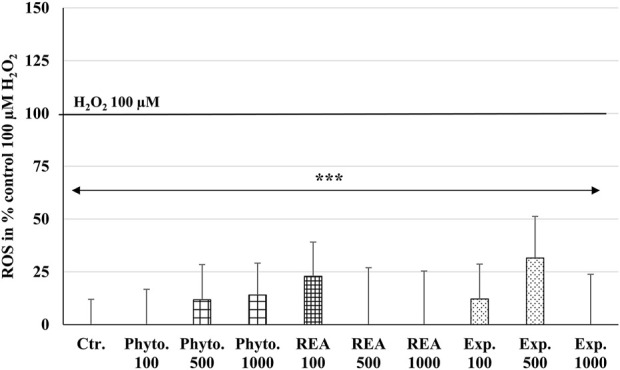
Effects of Phytohustil^®^ (Phyto.), its excipients (Exp.), or root extract of *A. officinalis* L (REA) on intracellular reactive oxygen species (ROS) production in HUVEC, using the cell-permeant reagent DCFDA (excitation/emission _495 nm/529 nm_). Values in % fluorescence units (RFU) = [(sample DCFDA _495 nm/529 nm_) x 100]/(untreated DCFDA _495 nm/529 nm_) are given as mean + SEM; significance calculated by *t*-Test is indicated as ***p ≤ 0.001 vs. H_2_O_2_ 100 µM control (100% ROS); *n* = 11 independent experiments.

**FIGURE 5:**
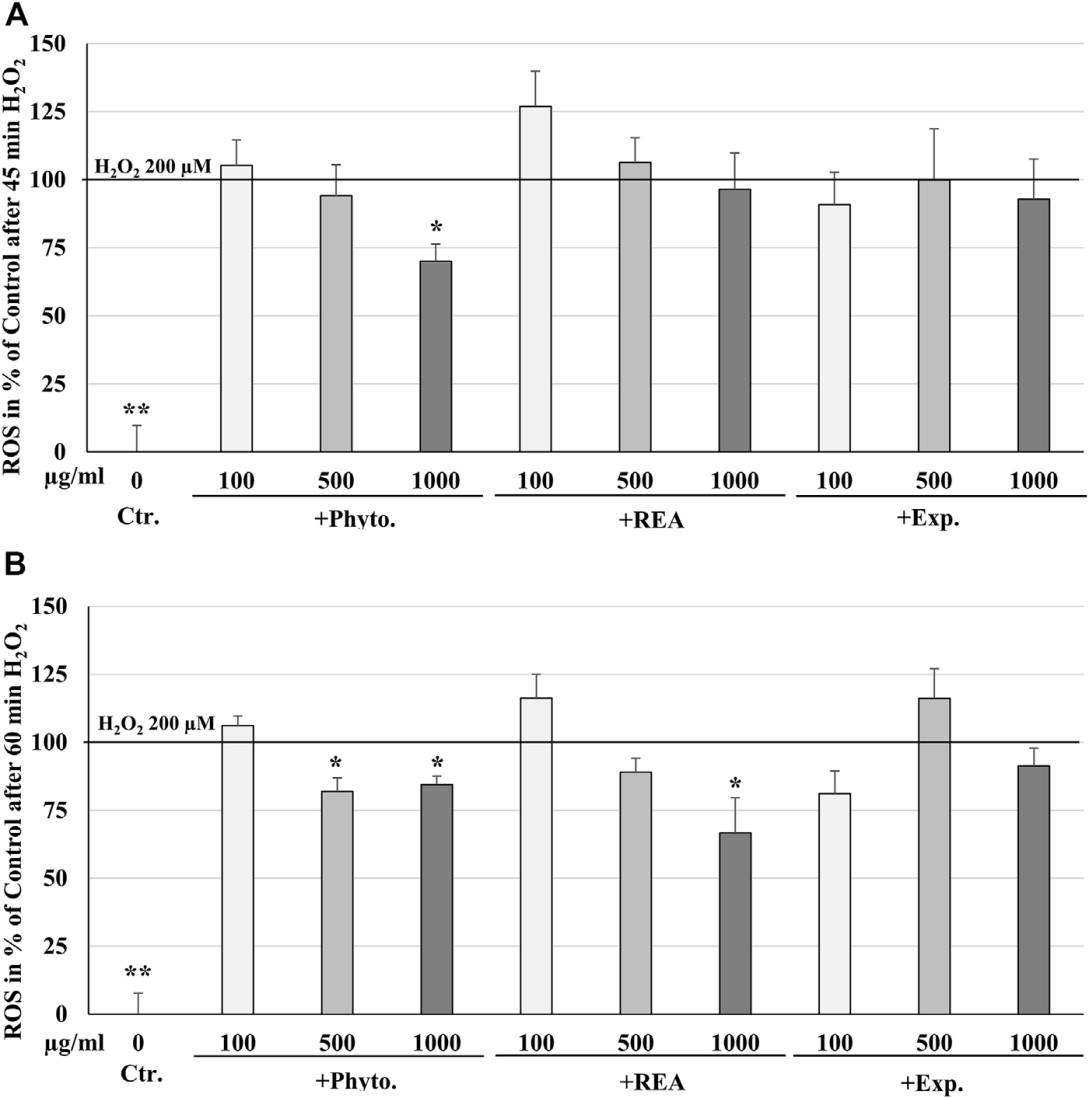
Protective effects of 24 h pretreatment of HUVEC with Phytohustil^®^ (Phyto.), its excipients, or root extract of *A. officinalis* L (REA) against H_2_O_2_–induced intracellular reactive oxygen species (ROS) production after 30 min **(A)** or 60 min **(B)** H_2_O_2_ treatment, using the cell-permeant reagent DCFDA (excitation/emission _495 nm/529 nm_). Values in % fluorescence units (RFU) = [(sample DCFDA _495 nm/529 nm_) x 100]/(untreated DCFDA _495 nm/529 nm_) are given as mean + SEM; significance calculated by *t*-Test is indicated as *p ≤ 0.01, **p ≤ 0.01 vs. H_2_O_2_ 200 μM; *n* = 4 independent experiments.

ROS are produced by living cells as a normal cellular metabolic byproduct, generated during mitochondrial oxidative activity. Under stress conditions, cells produce more ROS. Oxidative stress occurs when ROS rise above the antioxidant defense capacity because of a decrease in the intracellular antioxidant capacity or an increase in ROS levels. The incubation of HUVEC with 100, 500, or 1,000 µg/ml Phytohustil^®^, its excipients or REA, did not affect the intracellular ROS level compared to the untreated control (Figure 4). Whereas treatment with H_2_O_2_ (100 µM) significantly (*p* ≤ 0.001) increased the intracellular ROS production by 100% compared to untreated control (Figure 4). After 24 h pre-treatment of HUVEC with 1,000 µg/ml Phytohustil^®^ and additional 45 min incubation with 200 µM mM H_2_O_2_ the ROS production was significantly (*p* ≤ 0.05) inhibited by 30.0% (Figure 5A) and after 60 min by 18.1% (500 µg/ml) or 15.6% (1,000 µg/ml) compared to 200 µM H_2_O_2_ (Figure 5B). Interestingly, after pretreatment of HUVEC with 1,000 µg/ml REA and an additional 60 min, 200 µM H_2_O_2,_ the ROS production was significantly (*p* < 0.05) inhibited by 33.4% compared to 200 µM H_2_O_2_ (Figure 5B).

### Effects of Phytohustil^®^, its excipients or REA on the migratory capacity of human umbilical vein endothelial cells

**FIGURE 6:**
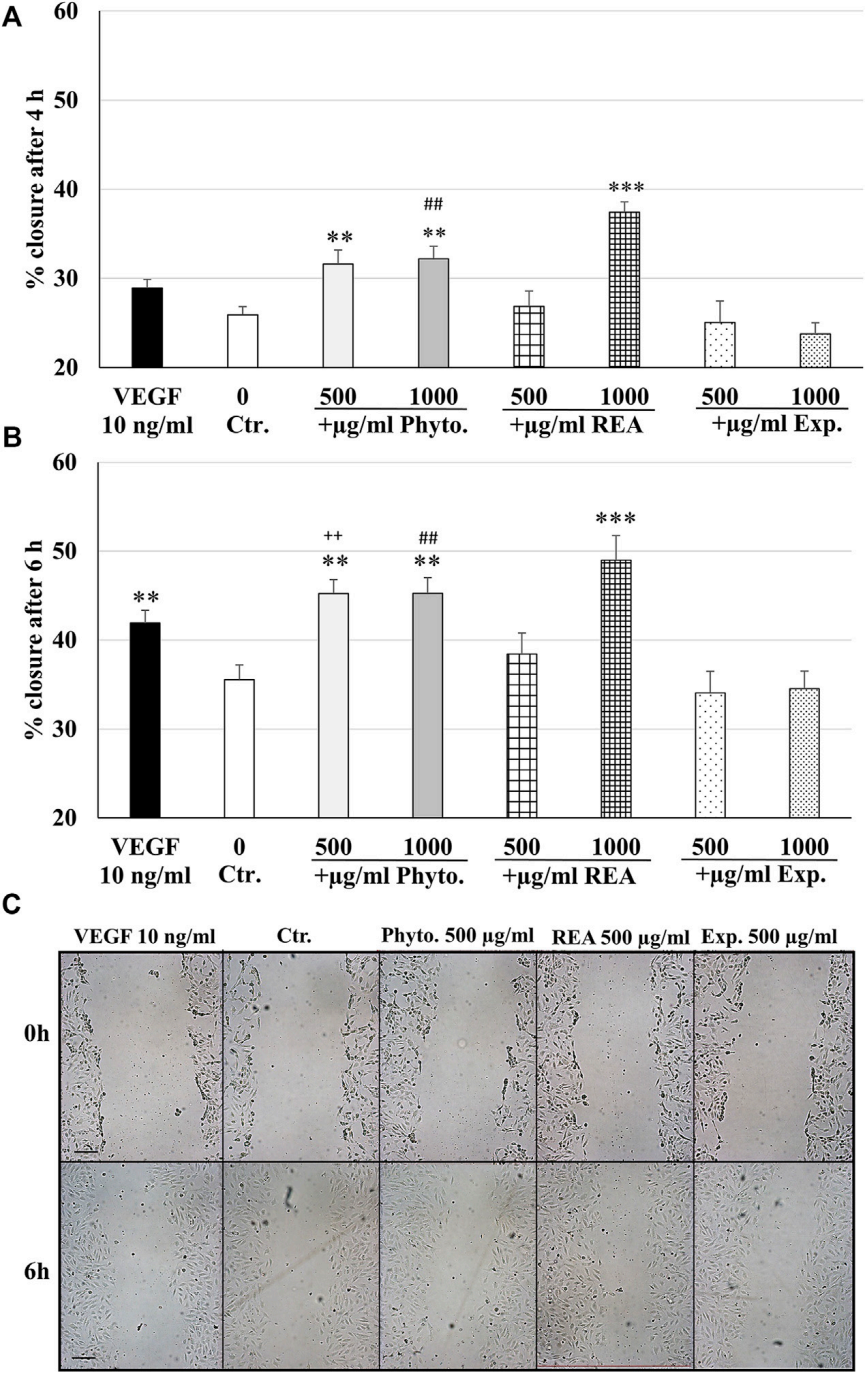
Stimulatory effects of Phytohustil^®^ or REA on the migratory capacity of HUVEC. Quantification of cell migration of HUVEC after scratching. HUVEC were treated with Phytohustil^®^ (Phyto.), its excipients (Exp.), REA for 4 h **(A)** or 6 h **(B)** or with medium alone (untreated control). Vascular Endothelial Growth Factor (VEGF) was used as a positive control of migration. The results are displayed in % of scratch closure as mean + SEM; significance calculated by *t*-Test is indicated as **p ≤ 0.01, ***p ≤ 0.001 vs. untreated control; Phyto. 500 µg/ml vs. Exp. ^++^ *p* ≤ 0.01; Phyto. 1,000 µg/ml vs. Exp. ^##^ *p* ≤ 0.01. **(C)** Representative images of the wound closure after 0 h and 6 h; *n* = 4 independent experiments. Scale bar: 100 µm.

We investigated the migratory capacity of HUVEC after treatment with Phytohustil^®^, its excipients, or REA using the scratch assay. The results indicate that the treatment (4 h) of HUVEC with 500 or 1,000 µg/ml Phytohustil^®^ significantly stimulated the wound closure by 5.7%, 6.3% (*p* ≤ 0.01) and incubation with 1,000 µg/ml REA yielded a significant increase (*p* ≤ 0.001) by 11.5% increased wound closure compared to the untreated control (Figures 6A,C). Interestingly, treatment (4 h) of HUVEC with 1,000 µg/ml Phytohustil^®^ significantly (*p* ≤ 0.01) stimulated the migratory capacity by 8.4% compared to the excipients (Figures 6A,C). Treatment (6 h) of HUVEC with 500 or 1,000 µg/ml Phytohustil^®^ significantly stimulated the wound closure by 9.7% (*p* ≤ 0.01) and after incubation with 1,000 µg/ml REA by 13.5% (*p* ≤ 0.001) compared to the untreated control (Figures 6B,C). When compared to the excipients, treatment of HUVEC with 500 or 1,000 µg/ml of Phytohustil^®^ significantly (*p* ≤ 0.01) stimulated the wound closure by 11.2% or by 10.7% (Figures 6B,C). VEGF (10 ng/ml), taken as a positive control for migration, increased significantly by 6.4% (*p* ≤ 0.01) the wound closure, however, only after 6 h treatment compared to the untreated control (Figures 6A–C).

## Discussion

*Althaea officinalis* L., commonly known as marshmallow, has been widely used since ancient times because of its healing properties, e.g., for treating the irritation of the mouth or throat and associated dry cough and against symptomatic gastrointestinal discomfort (European Medicines Agency. 2016). REA is commercially available as a drug, i.e., Phytohustil^®^ cough syrup.

In this study, we have performed *in vitro* experiments using primary endothelial cells, i.e., HUVEC, which have been commonly used for physiological and pharmacological investigations, as a model system to study angiogenesis. We found that Phytohustil^®^ increased the viability of HUVEC, whereas REA or its excipients alone did not. This could be interpreted as a synergistic effect between REA and the excipients, both components of Phytohustil^®^.

A characteristic of the mucosal tissue is its wet environment. Under these conditions, it has close contact with multiple and diverse typical commensal microorganisms and pathogens, which can invade and produce pro-inflammatory responses after a mucosa lesion. Purified marshmallow polysaccharides showed *in vivo* immune-activating effects (Wagner, 1990). In this context, we have recently published data on the anti-inflammatory properties of Phytohustil^®^ on human macrophages. We have shown that Phytohustil^®^ and the REA, but not its excipients inhibited the release of both TNF-α and IL6 by LPS-activated macrophages (Bonaterra et al., 2020). These findings corroborate the anti-inflammatory and possible immunomodulatory properties of the REA as published by others, however, in neutrophils (Scheffer et al., 1991).

Here we show that pretreatment of HUVEC with Phytohustil^®^ or REA inhibits the LPS-induced IL6 release and, therefore, corroborates the anti-inflammatory properties of REA at levels comparable to 25 µM diclofenac. These anti-inflammatory effects of the commercially available product Phytohustil^®^ and its main component REA on HUVEC are novel. Interestingly, we cannot detect IL-1β or TNF-α after LPS stimulation in our experimental set by HUVEC to investigate anti-inflammatory effects. The lack of proinflammatory stimulation by LPS of the cytokines IL-1B and TNF-α may be because HUVECs are proinflammatory stimulated by exogen IL-1β and TNF-α, instead of being released by HUVEC, according to other authors (Makó et al., 2010). Moreover, intracellular ROS homeostasis is essential in maintaining normal cellular physiology and integrity. We and others have shown that REA has chemical antioxidant properties (Sadighara et al., 2012), stimulates immune defense mechanisms in human BV-173 leukemic cells (Benbassat et al., 2014) as well as that Phytohustil^®^ and REA may protect against intracellular ROS increase in human macrophages (Bonaterra et al., 2020). Therefore, we have performed experiments to investigate the potential protective properties of Phytohustil^®^ or REA, against H_2_O_2_ -induced cytotoxicity and intracellular ROS production in HUVEC. We found an inhibition of H_2_O_2_ -mediated decrease of the viability when HUVEC were pretreated with Phytohustil^®^. This protective effect is shown for the first time. In this context, we also found that Phytohustil^®^ and REA reveal protective effects against the increase of harmful intracellular ROS, which was induced by H_2_O_2_ treatment of HUVEC. Thus, for the first time, we demonstrate that Phytohustil^®^ and REA can inhibit the damaging effects of oxidative stress on HUVEC and may positively influence wound healing processes through this pathway.

High molecular weight hyaluronic acid of REA exerts many effects on the tissue, such as activating the migration of leukocytes as monocytes/macrophages (Sendker et al., 2017). Induction of the production of growth factors by epithelial cells, proliferation, differentiation, and migration are also stimulating properties of these REA polymers and seem beneficial for tissue regeneration (Heldin, 2003; Sendker et al., 2017). Most recently, we have shown that REA can activate the migration of human macrophages (Bonaterra et al., 2020). This property may be associated with a chemoattractant activity of Phytohustil^®^. The REA induction of migration by macrophages into the injured and inflamed mucosa may have importance for the resolution of the inflammation and, consequently, wound healing (Chanput et al., 2010; Bonaterra et al., 2020). Using an *in vitro* scratch assay, we show that Phytohustil^®^, and its active ingredient REA, but not the vehicle with excipients, activate the migration of HUVEC. We found a similar mechanism that may explain our results related to the pro-migratory effect on macrophages after treatment with Phytohustil^®^ (Bonaterra et al., 2012). These results reveal evidence for the stimulating properties of vascularization and may be interpreted as a positive repair effect against mucosal injury, e.g., elicited by a dry cough. Additionally, with the stimulation of the endothelial cell migration by Phytohustil^®^ and REA might be to be expected that *in vivo,* this effect could be significant for repairing the innervation, which has probably been injured after a lesion of the oral mucosa. As described by others, this may be an additional property of the endothelial cells during the revascularization of the damaged mucosa after injury (Grasman et al., 2017).

## Conclusion

Our data prove that Phytohustil^®^ and REA have anti-inflammatory properties and protect vascular endothelial cells against oxidative stress and H_2_O_2_-induced cytotoxicity. In addition, Phytohustil^®^ improved the migratory capacity of HUVEC as an *in vitro* model of the vascular endothelium. The anti-inflammatory effects of Phytohustil^®^ or REA were similar to the anti-inflammatory drug diclofenac. These protective and stimulating features may support the therapeutical effects of Phytohustil^®^ and is suggested to be also beneficial in patients during the treatment of laryngopharyngeal irritated mucosal membranes, as well as to be appropriate for symptomatic treatment of a dry cough.

## Data Availability

The raw data supporting the conclusion of this article will be made available by the authors, without undue reservation.
